# Dipping Process Characteristics Based on Image Processing of Pictures Captured by High-speed Cameras

**DOI:** 10.1007/s40820-014-0012-6

**Published:** 2014-12-02

**Authors:** Junhui Li, Yang Xia, Wei Wang, Fuliang Wang, Wei Zhang, Wenhui Zhu

**Affiliations:** School of Mechanical and Electronical Engineering and State Key Laboratory of High Performance Complex Manufacturing, Central South University, Changsha, 410083 People’s Republic of China

**Keywords:** Dipping acceleration, Dipping speed, Dipping time, Viscosity, Image processing

## Abstract

The dipping process was recorded firstly by high-speed camera system; acceleration time, speed, and dipping time were set by the control system of dipping bed, respectively. By image processing of dipping process based on Otsu’s method, it was found that low-viscosity flux glue eliminates the micelle effectively, very low speed also leads to small micelle hidden between the bumps, and this small micelle and hidden phenomenon disappeared when the speed is ≥0.2 cm s^−1^. Dipping flux quantity of the bump decreases by about 100 square pixels when flux viscosity is reduced from 4,500 to 3,500 mpa s. For the 3,500 mpa s viscosity glue, dipping flux quantity increases with the increase of the speed and decreases with the increase of the speed after the speed is up to 0.8 cm s^−1^. The stable time of dipping glue can be obtained by real-time curve of dipping flux quantity and is only 80–90 ms when dipping speed is from 1.6 to 4.0 cm s^−1^. Dipping flux quantity has an increasing trend for acceleration time and has a decreasing trend for acceleration. Dipping flux quantity increases with the increase of dipping time, and is becoming saturated when the time is ≥55 ms.

## Introduction

Flip-chip technology has been widely used in high-performance and high-density microelectronics packaging [1–8] due to shorter possible leads, lower inductance, higher frequency, better noise control, smaller device footprints, and a lower profile, such as smart card, laser emitting diode, and surface-acoustic-wave filter in telecom applications [9–13]. Flux coating is one of the key processes during flip-chip packaging; flux is applied on the solder bumps or substrate to remove the oxides, pre-bonding flip chip on the substrate before reflow, and increases the wettability of the solder bump and improve assembly reliability [14–16]. Usually, flux coating can be achieved through dipping flux, ultrasonic flux, printing flux, and so on [17]. Chip to wafer (C2W) flip-chip bonding is more suitable at present for IC integration with expected process flexibility [18]. A flux dipping method was applied to C2W bonding [19]. Manna [20] and Nyamannavar et al. [21] reported that effect of fluxing chemical on wire surface by hot dip process and Heat Flux Transients at Solder/Substrate interface. The dipping quantity of flip-chip bumps affects the flip-chip bonding effect. Little flux can result in unbonding, excessive flux influence reliability of flip-chip.

However, from the literature, studies on the dipping process are quite lacking. Expected to develop new methods to observe the dipping process, adopt new ways to express the dipping quantity of tiny bumps and precisely control the dipping quantity.

In the paper, flux viscosity, dipping acceleration, dipping time, and flux stable time were firstly investigated using high-speed camera system and Matlab data processing. Based on the experimental images and data, the dipping process is discussed further.

## Experiment

**Fig. 1:**
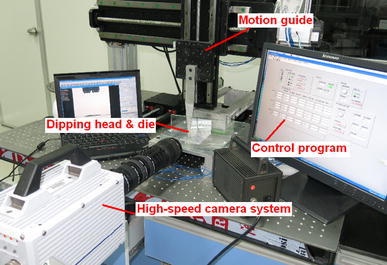
The dipping experimental bed and the high-speed camera system

The dipping experimental bed is shown in Fig. 1 which includes dipping head, motion guide, computers, and high-speed camera system. Type of motion control card is SMC2410B offered by Shenzhen Leadshine Technology Co., Ltd. Model of servo motor is MHMD042G1U offered by Panasonic Corporation, it is an AC servo motor. Its rated speed is 3,000 r min^−1^ and encoder resolution is *r* = 10,000 p r^−1^. Servo driver was matched with servo motor and have chosen Panasonic MINAS A5B series, model was MBDHT2510E. Motion guide is used to move up and down to achieve flux dipping.

**Fig. 2:**
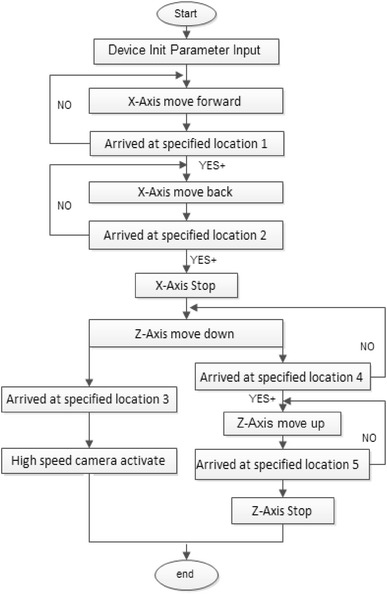
The flow chart of control system and image acquisition system

The control program was used in LabVIEW8.6 to integrate motion control system and image acquisition system. The flux container moved back and forth on the plate driven by *X*-axis. Flux was transferred into the flux groove through the movement of the container. The die was immerged into the groove and coating some amount of flux. When the die moves at a specified location driven by *Z*-axis, the high-speed camera gets activated and records the whole dipping progress. According to the dipping process, the flow chart of the main program is shown in Fig. 2.

**Fig. 3:**
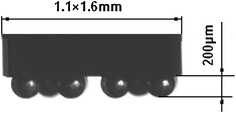
Flip-chip die

The parameters of dipping process are controlled by motion controller. Acceleration, acceleration time, speed and dipping time can be adjusted directly in the program interface. The dipping process was recorded quickly using FASTCAM-SA1.1 high-speed camera system. Shooting frequency was set to 2,000 frames per second, and the resolution is 1,024 × 1,024 pixel. The size of dipping die is 1.1 × 1.6 mm and 10 bumps, the diameter of bump is 267 μm, and the height of bump is 200 μm as shown in Fig. 3.

## Results and Discussion

**Fig. 4:**
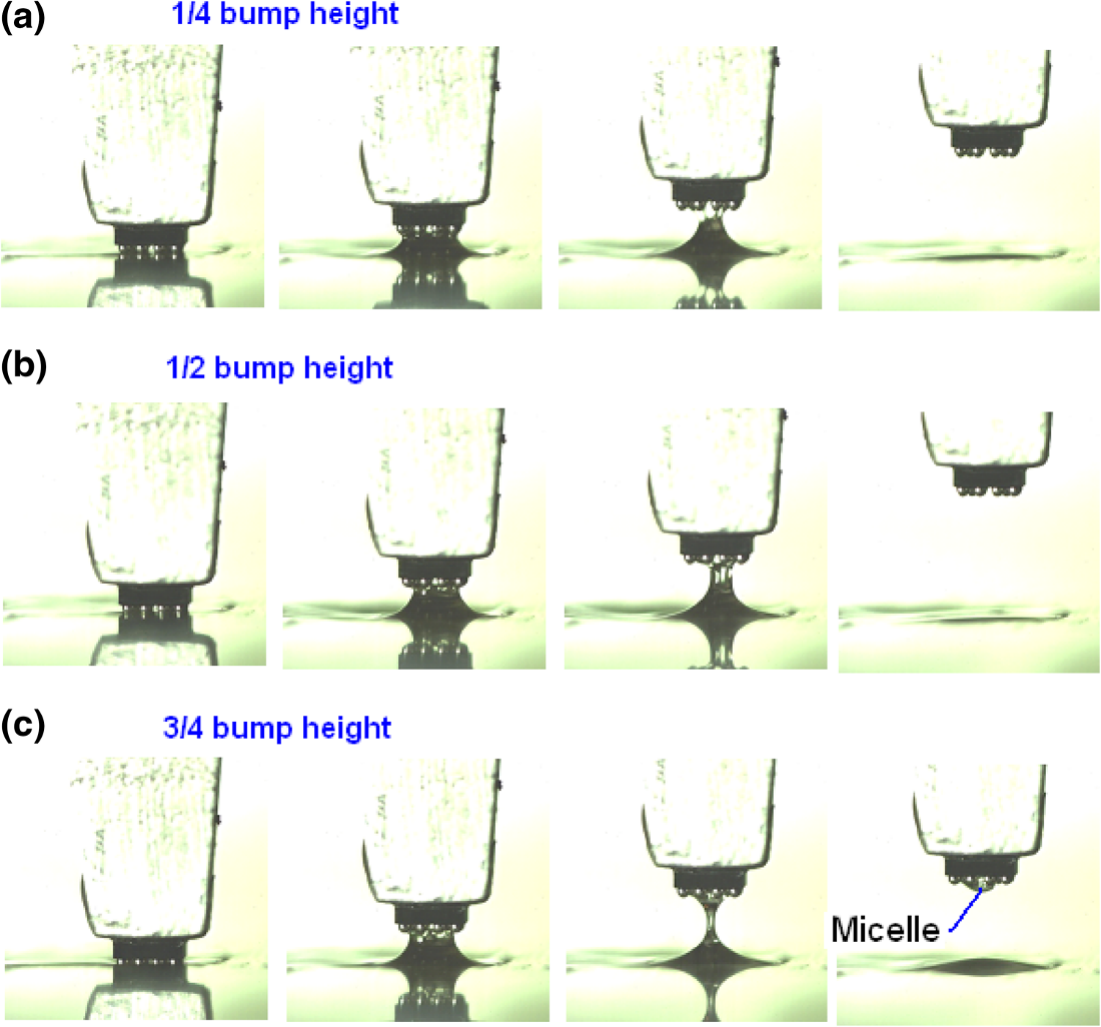
Sequence images of dipping process of 1/4 bump height from **a**, Sequence images of dipping process of 1/2 bump height from **b**, Sequence images of dipping process of 3/4 bump height from **c**

To determine the appropriate dipping depth, dipping depths were set at 1/4, 1/2, and 3/4 bump height. The sequence images of dipping process of 1/4, 1/2, and 3/4 bump height are shown in Fig. 4. It shows that 3/4 dipping depth leads to a micelle. A lighter dipping depth should be chosen in order to avoid micelles. So, dipping height was set to 1/4 bump height in the experiment. Viscosity, speed, acceleration & acceleration time, and dipping time are, respectively, tested.

### The Influence of Flux Viscosity on Dipping

In order to compare the dipping effects of different viscosities, dipping experiments of 4,500 and 3,500 mpa s viscosity fluxes were carried out, respectively.

**Fig. 5:**
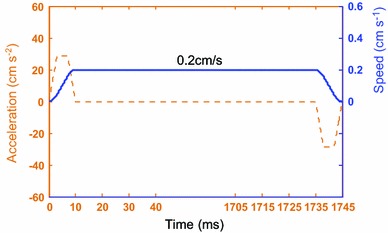
Real-time curve of 0.2 cm s^−1^ rising speed

**Fig. 6:**
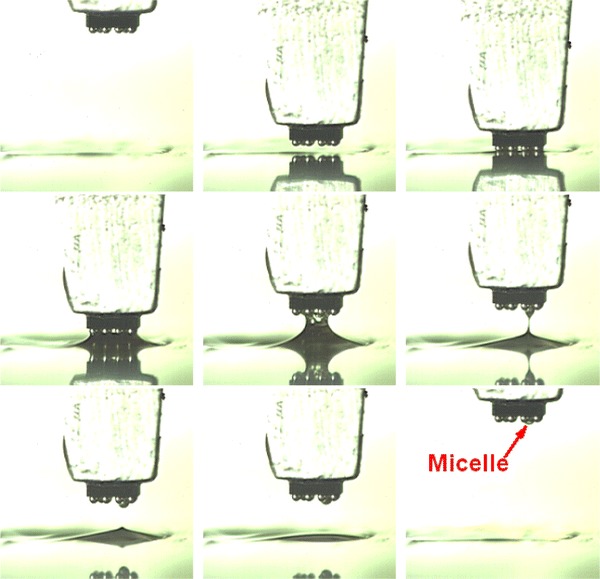
Sequence images of dipping process with the micelle for 4,500 mpa s viscosity flux

For 4,500 mpa s viscosity flux, the dipping process was recorded at 2,000 frames per second by high-speed camera system when rising speed is set to 0.2 cm s^−1^ as shown in Fig. 5, and sequence images of dipping process are shown in Fig. 6. It shows that the micelle is remained, and results in excessive flux.

**Fig. 7:**
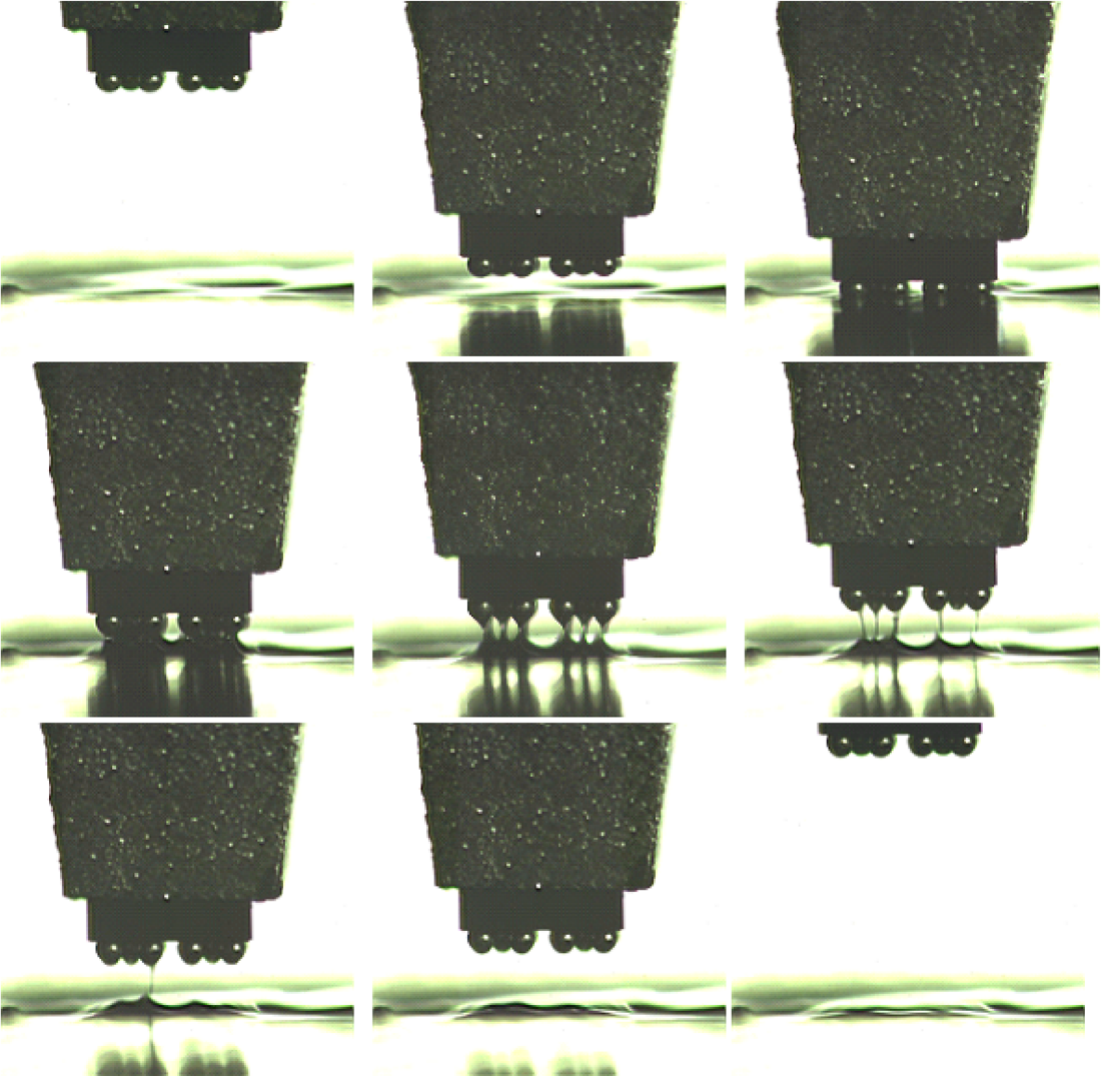
Sequence images of dipping process without the micelle for 3,500 mpa s viscosity flux

For 3,500 mpa s viscosity flux at the same rising speed, the dipping process is shown in Fig. 7. It shows that the micelle was effectively eliminated. The formation of micelles is inhibited by the low-viscosity flux.

**Fig. 8:**
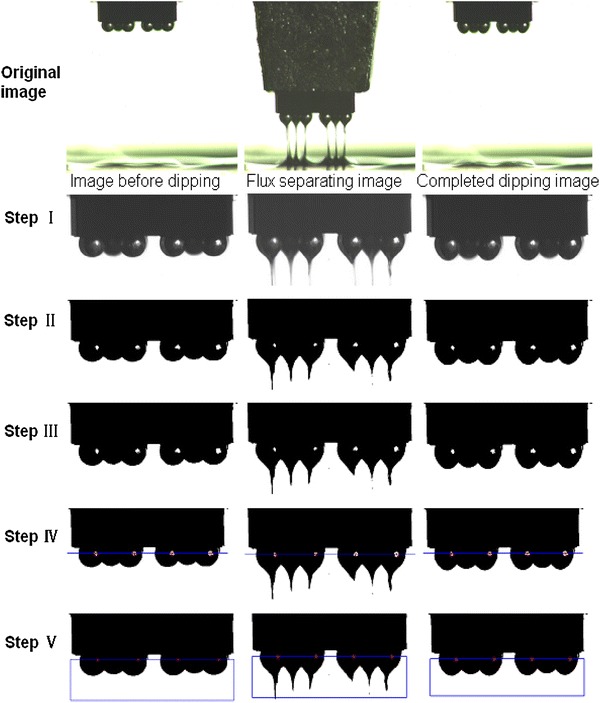
Image processing of dipping process images

To further research the influence of flux viscosity on dipping quantity of bumps, an image processing approach is applied to calculate the flux quantity using Matlab software as shown in Fig. 8. Through image processing of original images from step I to step V, the dipping quantity of bumps and flux flow process of dipping process can be obtained.

Step I is extracting image from the original images including image before dipping, flux separating image, and completed dipping image.

Step II is converting into a binary image using Otsu’s method. Otsu’s method is used to segment the image and extract image futures. Otsu’s method is an adaptive threshold determination method, and it is clustering based [22]. The basic principle of the method was shown as follows.

For a given image, suppose its pixels are represented in *L* gray levels (*1, 2,…, L*). Let threshold *T* divide gray levels into two parts *C*_0_ and *C*_1_, *C*_0_ consists of pixels with levels [*1,…,T*] and *C*_1_ consists of pixels with levels [*T* + *1,…, L*]. The number of pixels in *C*_0_ has a proportion of *w*_0_(*T*) in the whole image, and average gray level is *μ*_0_(*T*), the number of pixels in *C*_1_ has a proportion of *w*_1_(*T*) in the whole image, and average gray level is *μ*_1_(*T*). Let *μ* represents the mean level of the image; the between-class variance *σ*_2_(*T*) can be obtained by Eq. (1):

1 $$\sigma^{2}\left(T\right)=\omega_{0}\left(T\right){\left(\mu_{0}\left(T\right)-\mu \right)}^{2}+\omega_{1}\left(T\right){\left(\mu_{1}\left(T\right)-\mu \right)}^{2}.$$

Specifying the value of *T* from 0 to *L* sequentially, the number which makes *σ*_2_(*T*) has a maximum value is the threshold needed.

Step III is filling holes in a small area.

Step IV is identifying four holes’ center and fitting a straight line: firstly, finding out the centroid of reflective part on the solder bumps and, secondly, obtaining equation of the dividing line through *X*-direction coordinates of four centroids.

Suppose *D* is reflective area of the solder bump, *F* is a binary image, so the value of *F(x,y)* is 0 or 1. The equation to obtain centroid of reflective part on the solder bumps can be shown as Eq. (2) [23]:

2 $$\overset{¯}{x}=\frac{\sum_{D}xF(x,y)}{\sum_{D}F(x,y)},\;\overset{¯}{y}=\frac{\sum_{D}yF(x,y)}{\sum_{D}F(x,y)}.$$

*X*-direction coordinate of dividing line can be calculated by $x_{0}=\sum_{i=1}^{4}\bar{x_{i}}$ after four bumps centroids are obtained, and the function expression of the dividing line can be represented by *x* = *x*_0_.

Step*V* is filling holes and calculating bump area, dipping process area, and dipping bump area.

**Fig. 9:**
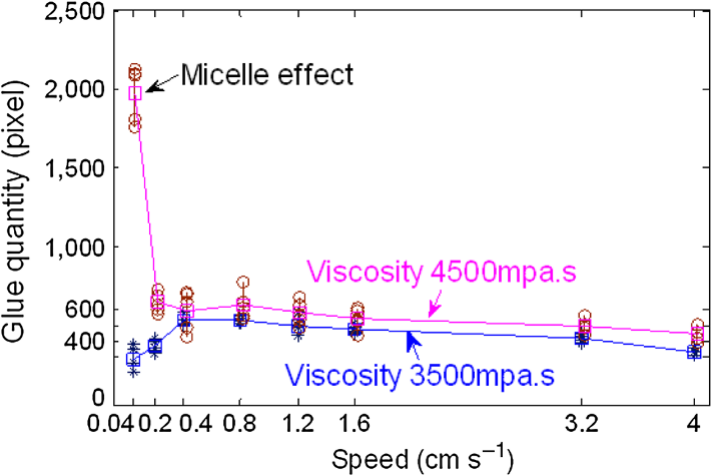
Flux dipping quantity comparison with 4,500 and 3,500 mpa s under the conditions of different speeds

In this way, difference of bump area and dipping bump area represents flux quantity, and difference of bump area and dipping process area shows flux flow process of dipping process. Flux dipping quantity is statistically shown in Fig. 9. It indicates that flux dipping quantity of 3,500 mpa s viscosity decreases 100 square pixels than 4,500 mpa s viscosity, and 0.04 cm s^−1^ low speed has a large flux quantity due to micelle effect. So, low-viscosity flux should be chosen to avoid excessive dipping and the formation of the micelle.

### The Real-time Characteristics of the Dipping

**Fig. 10:**
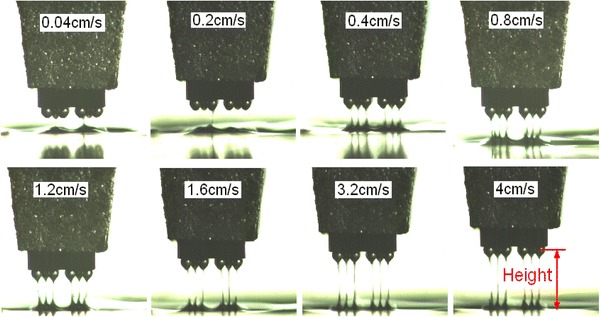
Separating height of the dipping process at different speeds

**Fig. 11:**
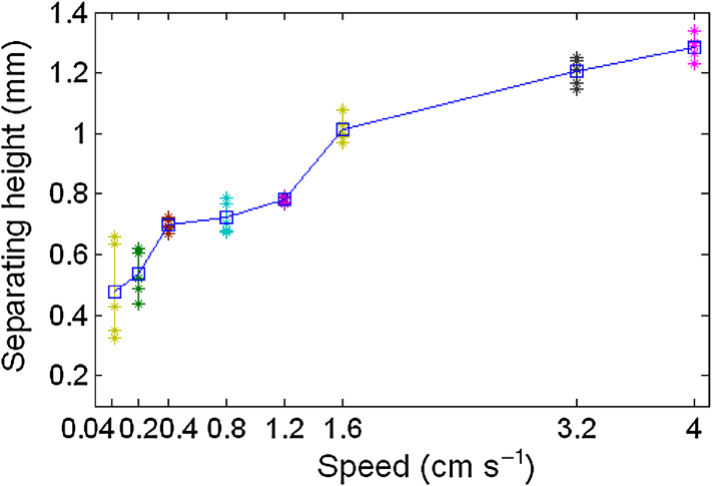
Statistical results of separating height at different speeds

**Fig. 12:**
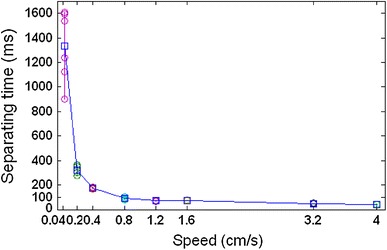
Statistical results of separating time at different speeds

Separating height of the dipping process was recorded as shown in Fig. 10, and its statistical results are shown in Fig. 11. The corresponding separation time is statistically shown in Fig. 12. It shows that flux separating height increases with the increase of speed, and the separating consistency of each bump is improved. Separating time decreases significantly with the increase of speed, and the separating simultaneity of each bump is improved.

**Fig. 13:**
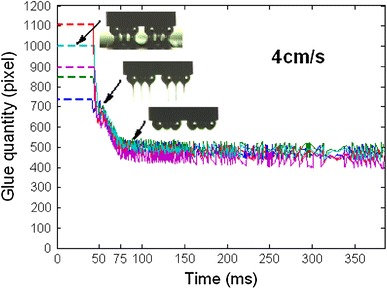
Real-time flux flow quantities of dipping process at 4 cm s^−1^ speed

**Fig. 14:**
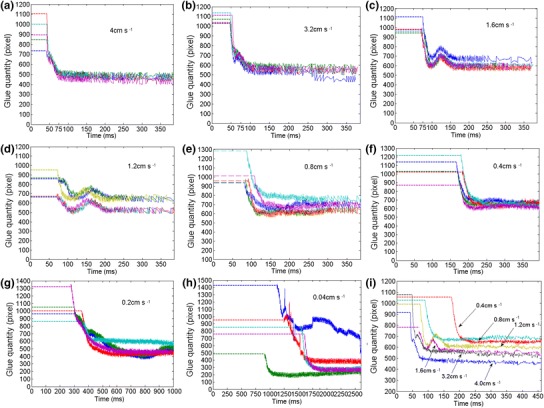
Real-time flux flow quantities of dipping process at different speeds

**Fig. 15:**
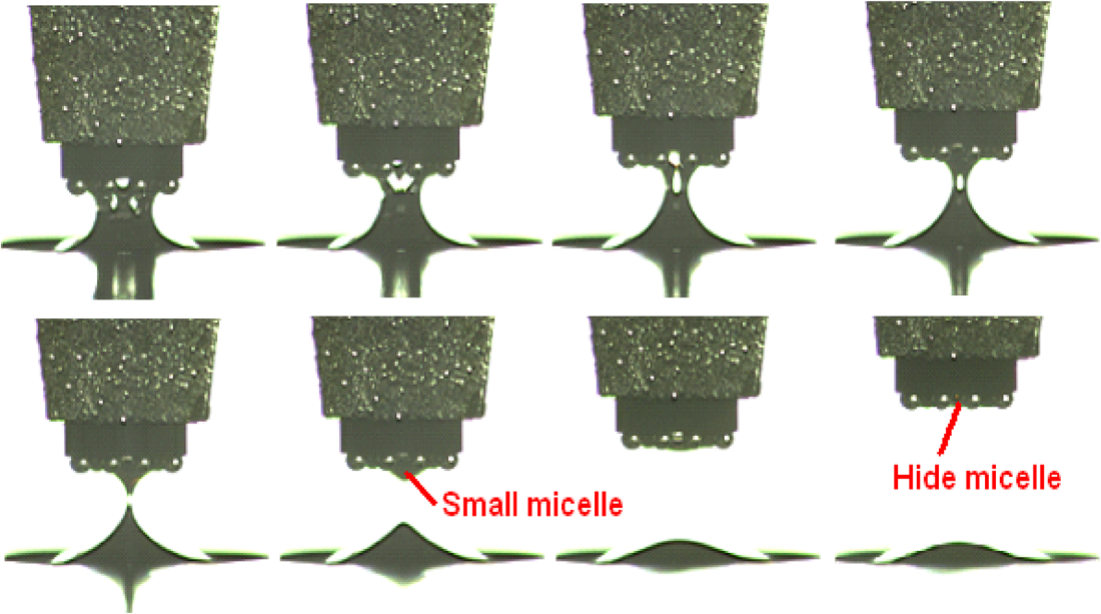
Small micelle formed and hidden between the bumps at the very low speed

Based on image processing from flux separating image to completed dipping image, flux flow quantities of dipping process are plotted in Fig. 13, where the experiment was carried out 5 times, respectively, at 4 cm s^−1^ speed. Before flux separation, dipping glue quantities were represented by dashed lines. It shows real-time characteristics. When the speed is 4, 3.2, 1.6, 1.2, 0.8, 0.4, 0.2, and 0.04 cm s^−1^, respectively, real-time characteristics of the dipping process are shown in Fig. 14a–h and i shows the average results at different speeds. Figure 14 shows that stable time and flux quantity vary with the speed. When the speed is very low (0.04 cm s^−1^), real-time curve has a big change as shown in Fig. 14h. This is because the small micelle were formed sometimes and hidden between the bumps as shown in Fig. 15 under the condition of the very low speed. The phenomenon disappeared when the speed is ≥0.2 cm s^−1^ for 3,500 mpa s viscosity flux.

**Fig. 16:**
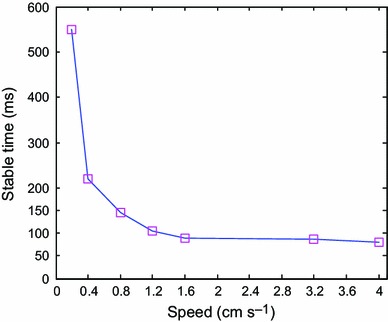
Stable time varies with the speed

The average stable time obtained from Fig. 14 is filled in Table 1 at different speeds and is plotted in Fig. 16. It shows that the stable time increases with the increase of the speed and is 80–90 ms when the speed is from 1.6 to 4 cm s^−1^. This will be a limit stable time.

**Table 1:** The average stable time at different speeds

Speed (cm s^−1^)	Stable time (ms)
4	80
3.2	86
1.6	90
1.2	105
0.8	145
0.4	220
0.2	550
0.04	1875

**Fig. 17:**
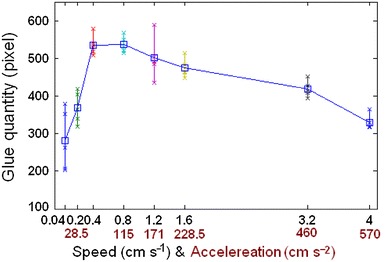
Dipping glue quantities varies with different speed (or acceleration)

Dipping glue quantities obtained from Fig. 14 are plotted, respectively, and averaged statistically in Fig. 17 at different speeds. It indicates that the curve of flux quantity increases with the increase of the speed in the beginning and decreases with the increase of the speed after the speed is up to 0.8 cm s^−1^. Therefore, the appropriate speed should be selected during dipping process in order to improve dipping efficiency and obtain appropriate glue.

### Acceleration & Acceleration Time Effect on the Dipping

In the above experiment, acceleration time is set to 10 ms; accelerations of the speed (0.2, 0.8, 1.2, 1.6, 3.2, and 4.0 cm s^−1^) are 28.5, 115, 171, 228.5, 460, and 570 cm s^−2^, respectively. The relationship between acceleration value and dipping flux quantity is shown in Fig. 17.

**Fig. 18:**
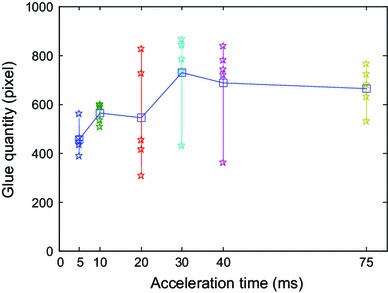
Dipping flux quantity varies with acceleration time

**Fig. 19:**
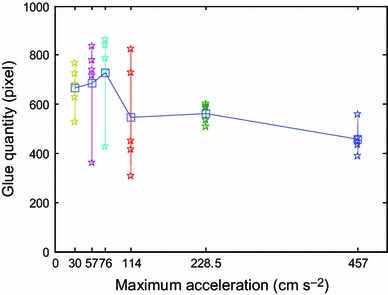
Dipping flux quantity varies with acceleration

For the same speed as 1.6 cm s^−1^, acceleration times were set to 5, 10, 20, 30, 40, and 75 ms, and the corresponding accelerations were 457, 228.5, 114, 76, 57, and 30 cm s^−2^, respectively. Dipping flux quantity varies with acceleration time and acceleration as shown in Figs. 18 and 19. It shows that dipping flux quantity has an increasing trend for acceleration time and has a decreasing trend for acceleration. In order to reduce the dipping flux quantity and improve the dipping efficiency, high acceleration and short acceleration time should be selected.

### Dipping Time Influence on the Dipping

**Fig. 20:**
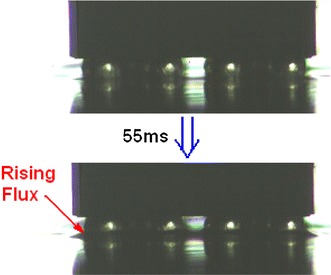
Flux glue rises along the edge of bumps for 55 ms dipping time

**Fig. 21:**
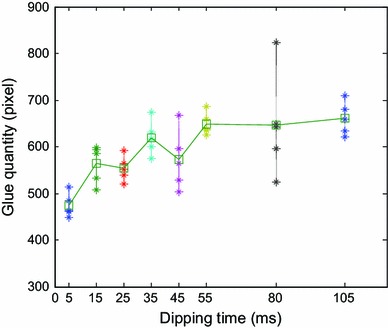
Dipping flux quantity of bumps varies with dipping time

Flux glue rises along the edge of bumps in glue pool due to surface tension effect of flux liquid as shown in Fig. 20. The longer dipping time is, the more the rising glue is. Rising flux will affect the final dipping quantity. Dipping flux quantity varies with dipping time as shown in Fig. 21. It shows that dipping flux quantity has an increasing trend with the increase of dipping time and becomes saturated value when dipping time is ≥55 ms.

## Conclusions

In conclusions, the parameters of the dipping process were analyzed in detail, including flux viscosity, speed, acceleration, acceleration time, and dipping time, and the images of high-speed camera system indicate dipping performance information by the image processing. The main findings are as follows:

1. The influence of flux viscosity on dipping is very obvious. Low-viscosity flux glue eliminates effectively the micelle. Dipping flux quantity of the bump decreases by about 100 square pixels when flux viscosity is reduced from 4,500 to 3,500 mpa s.
2. Real-time curve indicates the dipping process. The stable time of dipping glue decreases with the increase of the speed and has the same limit stable time as 80–90 ms when the speed is from 1.6 to 4 cm s^−1^. Dipping flux quantity increases with the increase of the speed in the beginning and decreases with the increase of the speed after the speed is up to 0.8 cm s^−1^.
3. Very low speed sometimes leads to the small micelle hidden between the bumps during dipping process, and the phenomenon disappearing when the speed is ≥0.2 cm s^−1^.
4. Dipping flux quantity has an increasing trend for acceleration time or has a decreasing trend for acceleration.
5. Dipping flux quantity increases with the increase of dipping time and is becoming saturated when the dipping time is ≥55 ms.

In summary, experimental results have provided important real-time data for developing dipping technology.
